# Supplementary material for: Does Tobler’s first law of geography apply to internet attention? A case study of the Asian elephant northern migration event
Source: PLoS One. 2023 Mar 1;18(3):e0282474. doi: 10.1371/journal.pone.0282474 (PMC9977022; doi:10.1371/journal.pone.0282474)
Supplement: Attachment — Submitted filename: Response to Reviewers.docx [file pone.0282474.s003.docx]

We are very sincerely grateful to the two reviewers for their valuable comments, which have greatly helped improve the paper's quality. The authors have carefully and conscientiously revised the paper based on the reviewers' comments, and the revisions are described below.

**Reviewer #1:** This study explored an interesting question that does the Tobler's first law of geography apply to cyberspace by using a good example, i.e., the event of elephant migration in southwest China. The manuscript is clearly structured and well written in English. The presented data, methods, and analysis sound reasonable. I like the topic of this study and it sheds a light on the relation of “the Tobler’s first law of geography” and “network attention in cyberspace”.

However, the main issue of this study is that the conclusions are drawn only from analysis toward the event of elephant migration in southwest China. Even though it is a good case for study this topic, the conclusions are still event specific. In other word, one would question if the conclusions drawn in this study are general and solid. Some suggestions are as follows.

**(1) The authors are suggested to add “a case study of *** event” in the title. And the event should also be mentioned in the abstract.**

**Answer：**According to the comment of the reviewer, we have revised the title to "Does Tobler's first law of geography apply to internet attention? A case study of the Asian elephant northern migration event".

In addition, we have also mentioned this event in abstract, as follows:

**Abstract:** One of the basic assumptions of spatial theory is formulated in Waldo Tobler's first law of geography: "everything is related to everything else, but near things are more related than distant things." However, as internet space is a complex virtual space independent of the real world, whether this law is applicable to things in the internet space remains to be explored in depth. Therefore, this study takes the event of Asian elephant northern migration as an example, attempts to investigate the issue of the applicability of Tobler's first law of geography to internet attention by integrating geographic methods such as spatial visualization, spatial correlation analysis, and Geo-detector. The results show that Tobler's first law of geography does not fully apply to internet attention, which does not decay with increasing distance. Geographical distance, within certain boundaries, is influenced by "identity" and "relevance", and still plays a large role in internet attention. However, once the boundaries are exceeded, the impact of geographic distance on internet attention is weakened by the intervention of influencing factors such as the degree of information technology, population, and the strength of news media publicity. Overall, the strength of news media publicity has the greatest impact on internet attention. And when it interacts with geographic proximity, it has the most significant effect on internet attention.

**(2) Any analysis or conclusion should be carefully expressed throughout the manuscript.**

**Answer：**According to the comment of the reviewer, we have checked the analysis and conclusions of the whole article. Some analyses and conclusions were optimized and refined accordingly to enable a more precise presentation. Detailed revisions can be viewed in the revised manuscript.

**(3) This limitation should also be emphasized in the Discussion section.**

**Answer：**According to the comment of the reviewer, we have emphasized the limitations of the study in the discussion. The detailed revisions are as follows.

Of course, there are some limitations of our study. First, this study analyzes the applicability of Tobler's first law of geography to internet attention, but the internet space is a complex virtual space that also includes other things beyond internet attention, and the study of these things needs to be further explored in the future. Second, this study is a case study, and more cases need to be added to verify it in the future. Finally, this study is an indirect discussion of the applicability of Tobler's first law of geography to internet attention based on the analysis of the spatial and temporal characteristics and influencing factors of internet attention. Future research can try to use quantitative models to establish more direct arguments for geospatial relationships.

**(4) Some studies have investigated the distance-decay effect in cyberspace, which are suggested to be mentioned in the manuscript. And therefore, this manuscript should emphasize or narrow down to “network attention” instead of “cyberspace”.**

*** Liu, Y., Wang, F., Kang, C., Gao, Y. and Lu, Y., 2014. Analyzing Relatedness by Toponym Co-Occurrences on Web Pages. Transactions in GIS, 18(1), pp.89-107.**

*** Krings, G., Calabrese, F., Ratti, C., & Blondel, V. (2009). A gravity model for inter-city telephone communication networks. Journal of Statistical Mechanics: Theory and Experiment, 7003.**

*** Gao, S., Liu, Y., Wang, Y., & Ma, X. (2013). Discovering spatial interaction communities from mobile phone date. Transactions in GIS, 17(3), 463-481.**

**Answer：**According to the comment of the reviewer, we focused the theme of the article on "internet attention" and revised the title to "Does Tobler's first law of geography apply to internet attention? A case study of the Asian elephant northern migration event" and revised the text accordingly.

In addition, we cite some literature investigating distance decay in cyberspace in the introduction of the paper. The details are as follows:

In recent years, empirical efforts attempting to contribute to this debate have utilized emerging data sources derived from location-based services[24-25]. Online social networks[26-31] and mobile communication networks[32-38] are drawing particular attention from researchers. For example, Liu proposed a method for capturing "relatedness between geographical entities" based on the co-occurrences of their names on web pages[26]. Yuan explored the spatial decay effect in mass media and location-based social media[31]. Gao discovered that a high correlation exists between phone users’ movements in physical space and phone-call interaction in cyberspace[34]. Kang identified the distance decay effect in intercity mobile communications of China using a subnet data set[35]. But few relevant studies have been conducted based on Internet attention. Therefore, this paper attempts to discuss this issue based on the perspective of internet attention.

24. Andris C. Integrating social network data into GISystems. International Journal of Geographical Information Science. 2016; 30(10): 2009-2031
25. Han SY, Tsou MH, Clarke KC. Revisiting the death of geography in the era of Big Data: The friction of distance in cyberspace and real space. International Journal of Digital Earth. 2017; 11(5): 451-469.
26. Liu Y, Wang FH, Kang CG, Gao Y, Lu YM. Analyzing relatedness by toponym co-occurrences on web pages. Transactions in GIS. 2014; 18(1): 89-107.
27. Grabowicz PA, Ramasco JJ, Gonçalves B, Eguíluz VM. Entangling mobility and interactions in social media. PLoS One. 2017; 9(3): e92196.
28. Kaltenbrunner A, Scellato S, Volkovich Y, Laniado D, Currie D, Jutemar EJ, Mascolo C. Far from the eyes, close on the web: Impact of geographic distance on online social interactions. Proceedings of the 2012 ACM workshop on workshop on online social networks. ACM, 2012.
29. Lengyel B, Varga A, Ságvári B, Jakobi Á, Kertész J. Geographies of an online social network. PLoS One. 2015; 10(9): e0137248.
30. Laniado D, Volkovich Y, Scellato S, Mascolo C, Kaltenbrunner A. The impact of geographic distance on online social interactions. Information Systems Frontiers. 2018; 20(6): 1203-1218.
31. Yuan YY. Exploring the spatial decay effect in mass media and location-based social media: A case study of China. Springer International Publishing, 2017.
32. Calabrese F, Smoreda Z, Blondel VD, Ratti C. Interplay between tele-communications and face-to-face interactions: A study using mobile phone data. PLoS One. 2011; 6(7): e20814.
33. Expert P, Evans TS, Blondel VD, Lambiotte R. Uncovering space-independent communities in spatial networks. Proceedings of the National Academy of Sciences. 2011; 108(19): 7663-7668.
34. Gao S, Liu Y, Wang Y, Ma X. Discovering spatial interaction communities from mobile phone data. Transactions in GIS. 2013; 17(3): 463-481.
35. Kang C, Zhang Y, Ma X, Liu Y. Inferring properties and revealing geo-graphical impacts of intercity mobile communication network of China using a subnet data set. International Journal of Geographical Information Science. 2013; 27(3): 431-448.
36. Krings G, Calabrese F, Ratti C, Blondel VD. Urban gravity: A model for inter-city telecommunication flows. Journal of Statistical Mechanics: Theory and Experiment. 2009; (07): L07003.
37. Lambiotte R, Blondel VD, De Kerchove C, Huens E, Prieur C, Smoreda Z, Van Dooren P. Geographical dispersal of mobile communication networks. Physica A: Statistical Mechanics and its Applications. 2008; 387(21): 5317-5325.
38. Onnela JP, Arbesman S, González MC, Barabási AL, Christakis NA. Geographic constraints on social network groups. PLoS One. 2011; 6(4): e16939.

**(5) The authors are suggested to explain the term “network attention” earlier in the manuscript. In addition, “Internet attention” sounds better than “network attention” as “network” has a very broad meaning.**

**Answer：**According to the comment of the reviewer, We replaced the term " network attention" with " internet attention". The first paragraph of the article explains "Internet attention". The details are as follows:

Since the 1990s, human society has gradually moved from the industrial age to the information age [1]. The rapid spread of the internet makes information dissemination no longer limited by time and space, and extensive information is spreading rapidly and instantly. The China Internet Development Report (2021) shows that the number of Chinese Internet users reached 989 million by the end of 2020, and access to all kinds of information has become increasingly prevalent. Traditional geographical laws such as "distance decay" and "diminishing benefits" are defined in this context as "spatial and temporal compression" [2], and pessimistic arguments including "death of distance" [3] and "the end of geography" [4] frequently emerge in academic circles. To respond to the challenges of the information age and explore the laws of space and time, the research framework of geography has been widened into sub-disciplines, including information geography [5], communication geography [6-7], telecommunication geography [8], cyberspace geography [9], virtual geography [10], and media geography [11]. The aim is to explore the characteristics and laws of the territorial system of human-land relations from a geographic perspective in the information age, thereby expanding the research scope of geography [12]. In this context, this study attempts to investigate the applicability of Tobler's first law of geography to internet attention (An indicator to measure the active search behavior of Internet users based on their subjective information needs, which characterizes the attention orientation of the public) in the hope of enriching and improving the theoretical perception of distance decay in the Internet era.

**(6) In the Discussion section, the discussion about “whether the traditional laws of geography are applicable to network space-time” should be put in the first place. The limitation of the conclusions should be emphasized (i.e., conclusions are drawn from the case study).**

**Answer：**According to the comment of the reviewer, We discuss the limitations of Tobler's first law of geography for things in cyberspace in the first paragraph of the discussion. In addition, the limitations of this study as a case study are emphasized in the last paragraph. The details are as follows:

Tobler's first law of geography states that everything is related to everything else, but near things are more related to each other. It emphasizes the key role played by distance in the connection between things. Therefore, in the material world, this law has been more widely applied because there is a real spatial distance between things in the material world. However, in the current internet era, the concept of distance seems to gradually blur or even disappear in the internet space. So, does Tobler's first law of geography still apply to things in the internet space? This question remains to be continuously explored and discussed. Therefore, this study attempts to discuss the applicability of Tobler's first law of geography to internet attention in internet space, using the Asian elephant northern migration event as a research case. It helps to answer the pessimistic arguments about the "death of distance" and "the end of geography" in the internet era and further deepens the understanding of Tobler's first law of geography.

......

Of course, there are some limitations of our study. First, this study analyzes the applicability of Tobler's first law of geography to internet attention, but the internet space is a complex virtual space that also includes other things beyond internet attention, and the study of these things needs to be further explored in the future. Second, this study is a case study, and more cases need to be added to verify it in the future. Finally, this study is an indirect discussion of the applicability of Tobler's first law of geography to internet attention based on the analysis of the spatial and temporal characteristics and influencing factors of internet attention. Future research can try to use quantitative models to establish more direct arguments for geospatial relationships.

**(7) A small mistake in Page 11: “In the west, the strength of media publicity (X11) and geographical proximity (X11) passed the significance test.” The geographical proximity is X12 instead of X11.**

**Answer:** According to the comment of the reviewer, we checked and calibrated the numbers of all the variables in the article.

Reviewer #2: This study aims to analyze the geographical characterize of media attention of certain event, specifically, focuses on whether the network attention following some geography law “The firs law of geography”, and further investigates the influence of geographical factors on attention. I have some comments as follow:

**1. The title of this manuscript is “Does Tobler's first law of geography apply to cyberspace? An exploration based on network attention”, However, the content is little related with the topic. There is no specific experiment to verify the first law of geography, authors only conduct some conclusions from several figures. Therefore, the content does not follow the title.**

**Answer:** As the reviewer noted, the original manuscript was not sufficiently focused on the theme of the article. Therefore, we have made some changes in the revised manuscript to further focus our research themes.

The research logic of this study is to first observe whether the closer the area to the event, the higher the internet attention is. Then, we applied the quantitative statistical method "Geo-detector" to further verify whether geographic proximity has a significant effect on internet attention at the scale of the full sample and sub-regional samples (Sub-regional samples differ in geographical proximity), given the combined effect of many factors. In the original manuscript, we did not explain the purpose of each part of the study, so we have explained the purpose of the study at the beginning of "3. Spatio-temporal characteristics" and "4. Factors Influencing the Spatio-Temporal Evolution" respectively in the revised manuscript to make the article more suitable for the topic. The details are as follows:

**3.Spatial and Temporal Characteristics**

In this part of the study, we try to observe whether the internet attention of a region is stronger when it is closer to the region where the event took place by summarizing the temporal and spatial characteristics.

**4. Factors Influencing the Spatio-Temporal Evolution**

In the spatial analysis above, we initially inferred that Tobler's first law of geography does not apply to internet attention and that the spatial divergence of internet attention is likely to be influenced by a combination of many factors. Therefore, in this part of the study, we introduce the method of Geo-detector to analyze the extent to which each factor, including geographic proximity, affects the spatial divergence of internet attention when many factors are combined.

In addition, we optimized the analysis of the results in the article. The focus on discussing the effect of geographic distance on Internet attention further strengthens the fit of the content of the study to the topic. The detailed changes can be found in the annotations of the revised manuscript.

Moreover, the number of the geographic proximity variable in the original manuscript was X12, and in the revised manuscript we adjusted the number of geographic proximity to X1 to be able to emphasize the topic more.

**Table 3. Index system of influencing factors of internet attention**

|  | **Number** | **Detection factor** | **Representative Indicator** | **Property** |
| --- | --- | --- | --- | --- |
| **Geographical location** | X_1_ | Geographic proximity | Shortest land transportation distance to Yunnan Province (km) | - |
| **Regional development** | X_2_ | Economic development level | GDP per capita (yuan) | + |
|  | X_3_ | Industrial development level | Gross domestic product of primary, secondary and tertiary industries (billion yuan) | + |
|  | X_4_ | Urbanization level | Urban population to total population ratio (%) | + |
| **Ecological environment** | X_5_ | Forest coverage | Forest cover area to total area ratio (%) | + |
|  | X_6_ | Farmland coverage | Agricultural land area to total area ratio (%) | + |
|  | X_7_ | The number of nature reserves | Number of nature reserves (pcs) | + |
| **Demographic characteristics** | X_8_ | Population | Total regional population (million people) | + |
|  | X_9_ | Population age structure | Ratio of population aged 15-64 to total population (%) | + |
|  | X_10_ | Educational attainment | Ratio of population with education level above high school compared to total population (%) | + |
| **Network and publicity** | X_11_ | The degree of information technology | Internet penetration rate (%) | + |
|  | X_12_ | The strength of news media publicity | Baidu information concern index (-) | + |

Finally, we strengthen the discussion of the impact of geographic distance on internet attention in the "5.1 Discussion" section.

**2. The term ”network attention ” is easy to have ambiguity. The network usually refers to complex network. In fact, this study just refers to news attention.**

**Answer:** As the reviewer said, cyberspace is a complex space, and internet attention can not represent cyberspace, in fact, the object of this study should be "internet attention", so we changed the title from "Does Tobler's first law of geography apply to cyberspace? An exploration based on network attention" to "Does Tobler's first law of geography apply to internet attention? A case study of the Asian elephant northern migration event" in order to make the topic fit with the content of the study. Moreover, we explained the meaning of internet attention in the first paragraph of the article.

3. **From Figure 2, the six maps are same, it can not see the difference among these figures, which makes these maps no significant. Usually, I’m more concerned about the changing value of attention over time. For example, the attention in Province A and B are 100 and 1000 respectively on week 4, then the attention on week 7 are 400 and 1100. We can see that the changing value is 300 for Province A, while the changing value is only 100 for Province B. if we only visualize the total attention, it may miss the process of change.**

**Answer:**According to the comment of the reviewer, we have optimized the figures by adding values to be able to highlight the differences between the figures more. The details are as follows:

**Fig 2. Spatial representation of online attention in 31 provinces (municipalities directly under the Central Government and autonomous regions)**

**4. In impact factor, the population should play an important role on attention, the more population is, the more attention should be. The population density could not reflect this phenomenon.**

**Answer:**According to the comment of the reviewer, we changed the variable "population density" to "population", recalculated the results of the influencing factors, and made the corresponding adjustments throughout the text.

**Table 3. Index system of influencing factors of internet attention**

|  | **Number** | **Detection factor** | **Representative Indicator** | **Property** |
| --- | --- | --- | --- | --- |
| **Geographical location** | X_1_ | Geographic proximity | Shortest land transportation distance to Yunnan Province (km) | - |
| **Regional development** | X_2_ | Economic development level | GDP per capita (yuan) | + |
|  | X_3_ | Industrial development level | Gross domestic product of primary, secondary and tertiary industries (billion yuan) | + |
|  | X_4_ | Urbanization level | Urban population to total population ratio (%) | + |
| **Ecological environment** | X_5_ | Forest coverage | Forest cover area to total area ratio (%) | + |
|  | X_6_ | Farmland coverage | Agricultural land area to total area ratio (%) | + |
|  | X_7_ | The number of nature reserves | Number of nature reserves (pcs) | + |
| **Demographic characteristics** | X_8_ | Population | Total regional population (million people) | + |
|  | X_9_ | Population age structure | Ratio of population aged 15-64 to total population (%) | + |
|  | X_10_ | Educational attainment | Ratio of population with education level above high school compared to total population (%) | + |
| **Network and publicity** | X_11_ | The degree of information technology | Internet penetration rate (%) | + |
|  | X_12_ | The strength of news media publicity | Baidu information concern index (-) | + |

**5. A conclusion should be given in the manuscript.**

**Answer:**According to the comment of the reviewer, we have added a section "5.2 Conclusion" to explain the main conclusions of this study.

**5.2 Conclusion**

This study explores the applicability of Tobler's first law of geography to Internet attention using the Asian elephant northern migration event as a research case. We found that Tobler's first law of geography does not apply to internet attention, and the distance decay effect is not fully reflected in internet attention. Distance has an effect on internet attention, but this effect is often limited to a certain distance, and when a certain distance threshold is exceeded, the degree of influence of geographic proximity on internet attention is probably no longer significant due to the intervention of other influencing factors. Therefore, the influence of distance on internet attention is not decisive. The factor that really has a decisive influence on internet attention is the strength of news media publicity, and it has the greatest influence on internet attention when the strength of news media publicity interacts with geographic proximity.
